# The challenges and experiences of academics supporting psychological capital among students at the emergence of COVID‐19: A qualitative investigation of award‐winning educators at a British university

**DOI:** 10.1111/bjep.12565

**Published:** 2022-12-02

**Authors:** Roseanne Morris, Mark Hoelterhoff, Georgios Argyros

**Affiliations:** ^1^ University of Edinburgh Edinburgh UK

**Keywords:** academia, COVID‐19 in academia, higher education, psychological capital, strengths‐based approach

## Abstract

**Background:**

Studies show that student mental health has continued to deteriorate over the years. Developing strengths‐based approaches could aid educators in the development of Psychological Capital (PsyCap) and positive protective factors in students to support their mental well‐being and aid in their success; however, little is known of the subject experience of educators who attempt this.

**Aims:**

This study aims to understand the experience of award‐winning educators; both in their attempts to cultivate positive protective factors in students and in challenges to the pursuit of that goal during the shifting academic landscape at the emergence of COVID‐19.

**Sample:**

Six award‐winning educators from a British university.

**Methods:**

Participants were interviewed over video calls in this research design using semi‐structured interviews. Thematic analysis was used to analyse the data.

**Results & Discussion:**

The results showed two major themes; pressures for academics and strength‐based approached to cultivating PsyCap. These themes reflected that educators saw an urgent need for students to develop resilience as they struggle to handle subjective failure and that students struggle with imposter syndrome. The educators identified the challenges as feeling taken for granted, having unmanageable workloads along high expectations placed on them.

**Conclusion:**

COVID‐19 has added significantly to the workloads of educators and demonstrated students' need for resilience. This research identifies the experiences of educators trying to improve strengths‐based practice while identifying the challenges of pursuing that goal in the changing pedagogy post–COVID‐19.

## INTRODUCTION

Evidence suggests that there are high rates of depression and anxiety among students in higher education (Buchanan, 2012; Saleh et al., 2017; Syed et al., 2018). However, only a small percentage of these students receive treatment from university health services (Regehr et al., 2013). Thus, it is imperative to introduce sustainable practices at universities to safeguard students' mental health and cultivate positive well‐being in the academic community. This paper brings together two emerging areas in positive psychology and education; Psychological Capital (PsyCap) and Strengths‐Based Education (SB‐E). PsyCap is the amalgamation of positive psychological resources of hope, self‐efficacy, resilience and optimism (Luthans et al., 2007). SB‐E is an approach to education that encourages the cultivation and use of personal strengths to achieve goals (Lopez & Louis, 2009). By utilizing a strengths‐based approach, PsyCap can be cultivated in both students and educators alike, influencing students' retention and academic success and staffs' job satisfaction. This study aims to add to the understanding of cultivating protective or positive factors in students and exploring educators' needs in the pursuit of that goal by interviewing award‐winning educators.

Psychological Capital is a construct that is linked with positive well‐being and thriving (Luthns & Youseff, 2004). The four pillars of PsyCap are; hope, self‐efficacy, resilience and optimism. Initially a positive organizational behaviour construct, PsyCap was developed to invest in the blossoming and success of employees, rather than just the organization itself (Cerovic & Grudic Kvasic, 2016). In more recent years, the concept has been appearing in the academic sphere to explore how its cultivation in students can improve well‐being and performance in universities. High levels of PsyCap are linked with higher student retention, academic performance and happiness (Carmona‐Halty et al., 2019; Ortega‐Maldonado & Salanova, 2017).

Longitudinal research has shown PsyCap can be cultivated through the student‐teacher relationship (Carmona‐Halty et al., 2019). When educators place an emphasis on developing good relationships with their students, through showing interest in them and providing help and support, PsyCap in students flourishes (Carmona‐Halty et al., 2019). Luthans et al. (2016) demonstrated that PsyCap in students greatly increases student engagement levels. Further research indicated that the development of PsyCap was positively associated with students' current grade point average (GPA) and was a significant predictor of student's future academic success (Sweet & Swayze, 2020). Although the use of GPA to measure academic performance has been criticized as a narrow operationalization (York et al., 2015), there are numerous investigations that were found to have a positive relationship between PsyCap and GPA (Carmona‐Halty et al., 2021; Luthans et al., 2022; Noviati, 2018; Vanno et al., 2014). These studies addressed the validity of GPA as a measurement for academic performance by considering the complexity of this association. Specifically, it has been theorized that the impact PsyCap has on individuals' experience of positive emotions increases their levels of academic engagement, and their ability to self‐regulate their behaviours acts as a mediator to academic success as it allows them to effectively direct their PsyCap strengths towards greater academic performances (Carmona‐Halty et al., 2019; Luthans et al., 2022). In addition, PsyCap was shown to decrease dropout rates since it is linked with social and academic integration among peers and between faculty and students, which is another factor that increases academic performance (Sweet & Swayze, 2020).

Moreover, research suggests that increasing students' PsyCap can have beneficial effects beyond their academic achievements, influencing their overall quality of life and sense of well‐being (Ortega‐Maldonado & Salanova, 2017; Poots & Cassidy, 2020). The development of PsyCap has been particularly important over the last two years as the COVID‐19 pandemic resulted in higher prevalence rates of depression, anxiety and post‐traumatic stress symptoms among university students (Hekmat et al., 2021; Kumari et al., 2020; Salimi et al., 2021; Sultana et al., 2021). The deterioration of mental health has been even more pronounced in students who belong to minority groups (Wood et al., 2022). Evidence indicated that the construct of resilience, one of the pillars of PsyCap, was a negative predictor of depressive symptomatology in college students during COVID‐19 (Hagedorn et al., 2022). Analogously, three out of the four pillars of PsyCap (hope, resilience and optimism) were signified as predictors of well‐being during the pandemic, explaining almost 40% of the variance in scores (Prasath et al., 2021). Therefore, the promotion of PsyCap in higher education is essential. Novel teaching interventions and strength‐based education approaches could be implemented to increase students' positive psychological resources (Luthans et al., 2016; Sweet & Swayze, 2020).

Nevertheless, as Durrah et al. (2016) point out “educational institutions cannot improve the quality of their system without giving priority to their academic staff” (p. 183). Surveys from Times Higher Education (2015) show that many university staff are not thriving in their careers; they feel overworked and fearful of job stability. With the monetization of education and students essentially becoming ‘customers’, universities have effectively become businesses (Hemelt & Marcotte, 2011). Their staff, educators and scholars, are being required to fulfil the role of service provider, and meet higher demands for research, teaching and assessment feedback output, teamed with ever‐increasing class numbers (Bishop, 2015). Although there is a gap in the literature regarding the benefits of PsyCap in educators, cultivating these character strengths and skills could equip them with essential tools to support themselves and their students.

Despite the evidence showcasing the beneficial effects of PsyCap in students, there is no emphasis on the practices to cultivate its four pillars from the standpoint of the educator. However, strengths‐based approaches have demonstrated their capacity to cultivate positive well‐being in students. Strength‐based education is a student‐centred approach to education with the primary goal of developing students into confident, lifelong learners, whose work is imbued with a sense of purpose (Anderson, 2004). This approach begins with the educator developing and applying their own strengths as they help their students discover their strengths. Anderson (2004) highlights that this process is aided by educators keeping up to date with research in their fields, improving and developing their teaching methods and by establishing programme activities that cultivate strengths in their students. While the little empirical research on this approach proves to be promising, much more needs to be conducted to grasp its full potential.

The application of the five principles of strengths‐based education can aid in the cultivation of the four pillars of PsyCap in both students and educators (Lopez & Louis, 2009). The five key principles in the strengths‐based approach are; the measurement of strengths through strengths assessment; individualizing the learning experience; networking and building relationships creating social support; deliberate application of strengths; and intentional development of strengths. The implementation of psychoeducational strengths‐based interventions in an experimental group by Koydemir and Sun‐Selışık (2015) found enhancements in establishing social connectedness and positive relationships when compared with the control group. Moreover, this research demonstrated improvements in the regulation of emotions and increasing positive emotions, decision‐making and practising gratitude, all through cultivating their individual strengths (Koydemir & Sun‐Selışık, 2015). Each of these developments chimes with the underpinnings of psychological capital; hope, self‐efficacy, resilience and optimism.

A central implementation technique of strengths‐based education is through advising. Strengths‐based advising (SB‐A) in academia is where students engage with their academic advisors to discuss their strengths as an individual, how to cultivate them and how to use them (Schreiner & “Chip”Anderson, 2005). Research on the benefits of SB‐A in first‐year students found significantly higher retention rates in the first year and graduation in 4 years, higher levels of engagement and academic self‐efficacy in students who engaged with strengths‐based interactions with academic advisors (Koydemir & Sun‐Selışık, 2015; Passarelli et al., 2010; Soria et al., 2017; Zimmerman, 2013). Although they were not explicitly connected, the results showcase that SB‐A positively influenced students' levels of resilience and self‐efficacy, two of the pillars of PsyCap.

Therefore, research is needed to bridge the gap between cultivating psychological capital and strengths in students without adding to the already challenging workloads of educators. An emphasis must be placed on the development of strengths and psychological capital for the entire academic community, to allow everyone the opportunity to find their strengths and flourish. In addition, another gap that this investigation is addressing is the scarcity in the literature on PsyCap; a topic that has been predominantly studied in the United States. Interviewing award‐winning educators in the United Kingdom, who were recognized for their pedagogical approaches to teaching provides valuable insight into methods of cultivating psychological capital in students, obstacles educators come across in pursuing this, and potential solutions to aid in the nurturing of positive well‐being in students and educators alike. Specifically, this research investigation aims to:

1. Explore pressures in academia for students and educators
2. Identify the strengths‐based practices used by award‐winning educators that cultivate PsyCap in their students.
  2a. Explore the role PsyCap plays in the mitigation of negative and risk factors in mental health and success in students.

## METHODS

### Research design

Taking a narrative approach in this qualitative design, in‐depth interviews were utilized to glean insight into the perspectives and practices of participants (Elkatawneh, 2016). Such interviews provided flexibility in questioning, enabling the research team to accommodate any differences in participants' backgrounds while eliciting rich data (Guion et al., 2011). It also allowed for a comfortable ‘flow’ of conversation to keep participants comfortable and relaxed. A deductive approach was used for data analysis to allow themes to emerge. This research design facilitated the collection of high‐quality and rich data from participants (Elo & Kyngäs, 2008). Ahead of participant recruitment, ethical approval was sought and approved by the Research Ethics Committee. With much of the research on PsyCap taking place in the U.S.A., this study was conducted in Scotland to explore PsyCap on an international level.

### Participants

Thirteen members of the academic staff of the University of Edinburgh were invited to participate in the study, of which six participated. As noted in Fusch and Ness (2015), data saturation can be complicated to define due to both the research conditions and complexities of the available sample. Adhering to the concept of ‘information power’ put forward in the *Sample Size in Qualitative Interview Studies: Guided by Information Power* (Malterud et al., 2016), where the more information the sample holds relevant to the study, the fewer participants are needed, a total of 6 participants were recruited. Given the inclusion criteria of the participant's award‐winning teaching experience from a limited sample, 6 participants were justified as an acceptable amount to achieve information power in the data and feasible to interview and analyse given the project's constraints.

Each staff member received an Edinburgh University Students' Association (EUSA) Teaching Award between 2019 and 2020. These awards are based on student nominations, and the impact made on them by these educators. Students base their nominations on the educator's capacity to implement student feedback and use student‐centred learning, which are key aspects of cultivating PsyCap (McCune, 2021). Recognizing that these scholars had been acknowledged for excellence in their field of education, this study used purposive sampling to explore their perceptions and experiences as an educator on the subject of the student and academic staff experience. The invitation detailed an outline of the study, why they were invited to participate, that participation would involve a one‐hour interview over a video call and included the participant information and consent sheet. No follow‐up invitations were sent.

Six members of staff volunteered to participate. Five females and one male participated in this research. Each participant had obtained a PhD. Their experience working with students ranged from 5 to 25 years. The participants worked at various schools at the university; School of Chemistry (1), The Royal Dick School of Veterinary Studies (2), School of Biological Sciences (1), School of Literatures (1), Languages and Cultures (1) and College of Art (1). The participants were not affiliated with the research team's school. Further details on the participants' awards have not been discussed in an effort to maintain participant confidentiality.

### Materials

All participants received information sheets detailing the study's procedure, purpose, benefits and risks of participation, data protection, data withdrawal and voluntary participation. Contact information for the research team and external individuals from the School of Health Science who are separate from the study were also provided. Participants received a consent form detailing their voluntary participation.

The interview schedule consisted of 28 questions, which were based on the key principles of psychological capital, with a focus on 7 areas; background and demographic, personal awareness, strategy, challenges, impact, support and resources (Luthans et al., 2015). The formulation of the questions had theoretical underpinnings from relevant literature on strengths‐based education and perceptions of mental health and well‐being in the academic community, including that of students.

Listed are the topics discussed during the interviews based on existing literature (Lopez & Louis, 2009):

*Self‐awareness*—assessing self‐awareness in their role as an educator. This included how, when, and what they reflect on, their perceptions of their role as an educator.

*Strategies*—ascertain methods and approaches they use in their teaching and interactions with students.

*Challenges*—exploring approaches to challenges, what they consider to be challenging, and how they demonstrated resilience and personal strengths development in overcoming them.

*Impact*—gleaning insight into what impact they hope to have on students and to demonstrate skills in reflection. To gain insight into their views on the educator‐student relationship and the impact working with students has on them. To gain their perspective on student mental health and emotional well‐being from the role of the educator.

*Support and Resources*—exploring the status quo of mental health within the academic community, what support they receive, what key elements crop up that need addressing to ascertain what positive changes can be made to the system to improve the well‐being of academic staff and better equip them to be there for students.

### Data collection

In‐depth interviews were selected as the primary form of data collection for this study. Using semi‐structured interviews provided the flexibility to explore key themes and concepts on education and well‐being in academia with participants (Elkatawneh, 2016). All questions were open‐ended and additional questions were occasionally asked to either prompt the participant or to further explore a topic being discussed.

Video interviews were conducted using Microsoft Teams. This software was chosen as it contained direct links to the email and calendar software that are accessible to university staff and students. Interviews did not take place in person as the study was being conducted during the first COVID‐19 pandemic lockdown, June 2020, and necessary precautions were taken to protect participants and the research team. The interviews were audio‐recorded using an encrypted recorder. The audio recordings were stored in encrypted, password‐protected files until they had been transcribed. Once the interviews were transcribed and anonymized, the audio recordings were destroyed. All interviews took place over 7 working days. Interviews lasted between 35 and 75 min.

Interviews were transcribed verbatim by the research team from the audio recordings. A document of reflexivity was maintained while transcribing interviews to record recurring themes and to highlight/monitor the researcher's role during the interview. When all interviews were transcribed, each participant was contacted via email, offering them the opportunity to withdraw from the study. Two participants requested to view their interview transcripts. One transcript was returned with clarification on the number of years teaching. One participant alluded to some personal details they later wished to have redacted. There was no additional feedback from the other participants. At this point, all data were anonymized and the audio recordings were destroyed. All identifiable data in the transcripts were removed to ensure confidentiality of the data.

### Data analysis

A deductive approach was utilized for data analysis. The transcripts were broken down into the seven sections highlighted in the interview schedule and coded using the comment function on Microsoft Word. A coding software was considered; however, Microsoft Word was the preferred method for convenience to the research team. Thematic analysis was used following the six steps outlined by Braun and Clarke (2006); familiarization with data, generating initial codes, searching for themes, reviewing themes, defining and naming themes and producing the report. Each transcript was examined twice for coding.

Potential themes emerging from the data were noted. After coding the first three interviews, recurring codes became apparent. As the research team had curated the interview schedule, conducted and transcribed the interviews, there were elements of priming whereby the researchers consciously looked for specific codes in the data. It is important to note that the researchers were aware of specific elements such as demonstrating resilience in challenges and encouraging the individuality of students, in preparing for the study. With this in mind, reflections were recorded during the coding process to reduce personal bias. Once sufficiently familiar with the transcripts, the themes were created, revisited and finalized. Emerging themes were discussed with the study's supervisor to ensure they were fulfilling the aims of the study. A third‐party researcher who had not been involved in the data collection or initial analysis examined the themes as an additional measure of validity and robustness.

### Statement of reflexivity

While a deductive approach was taken to the analysis, there were times the researchers actively sought specific codes in the data. As the research team consisted of both students and staff at the university with personal experience with educators and university policy, there were instances where the researcher made assumptions based on their own experiences. Moreover, the researchers had preconceived notions that every school in the university would have the same culture and began leading the discussion in that direction at the beginning of the data collection process. After realizing that these assumptions were incorrect, the researchers developed a more open approach to each interview.

## RESULTS

The thematic analysis process following Braun and Clark's (2006) six‐step process that was applied to the transcripts deducted relevant key themes in the data informing the research aims of this study. The developed themes are; Pressures in Academia and Strengths‐Based Practices Cultivating PsyCap.

This study was conceived prior to the development of COVID‐19 and as such there was no way of knowing the impact it would have on the research. It came to light through the data collection process, in June 2020, that not only had the educators' personal lives been affected by the pandemic, but so too had their teaching style, student engagement and school's attitude to emotional well‐being. As such, the effect of COVID‐19 can be seen across all themes.

### Pressures in academia

How this system is set up is detrimental to people's mental health and wellbeing. And just because there are people who are resilient enough to get through it, doesn't mean that that should be the measure by which we judge everybody. So sometimes it's a bit overwhelming when you think about how mighty an issue it is. But you can have cultures in place and I think that can help. Participant 6 (P6)

This theme encompasses a number of elements (Table 1). The participants highlighted risk factors that students can experience and the responsibility that can weigh heavily on PhD students. For the educators themselves, it came to light that there was a sense of being taken for granted by the university, that the standards of excellence held by both themselves and their institution can be very demanding, and many felt ill‐equipped to handle some of the challenges they are presented with. With the event of COVID‐19, educators felt challenged with new workloads and the pressure of finding new and effective ways of engaging with their students.

**TABLE 1:** Example of codes for pressures in academia

Sense of identity tied to career	Does not support healthy work/life balance
Unique pressures in academia	Academic treadmill
Imposter syndrome	Unmanageable workloads
Expectations of excellence	Judging the individual on research

#### Risk factors for students

And sometimes I think that by, rather than trying to encourage them to cope as well as they can, but be satisfied with say slightly lower marks, then instead trying to take away things that students have said that they find difficult and then not giving them a realistic preparation for the fact that life is difficult, does them a bit of a disservice… Some students do struggle with the effects of stress and anxiety…, they're… high, high achievers… very… self‐critical, very hard on themselves. P5

A number of risk factors for students came to light from the data. Participants said that there is a fear of appearing as ‘stupid’ among their classmates and that the prospect of failure could have a significantly negative impact on a student's mental health. Two participants noted that it might not even be failing an assignment, but that they have failed by their own standards, highlighting the need for resilience in students;

Just as you mention resilience… one of the big issues we have is around students failing, whatever word that may mean… But we have some students that would never fail anything and then they go out in the profession and the first failure they have is an animal may die in surgery or something like that… P2

P2 highlighted the cruciality of developing resilience in students to sustain themselves, not only at university but also in their professional careers. This point was reiterated by another participant;

One of the things we really, really need in our students is resilience. One of the big problems in [their future]… profession is there's a very, very high dropout rate in the profession in quite early years. I can't remember the exact numbers but it's enormous, it's like something like 40 % have dropped out within the first 5 years. When you think about the investment they make both financially and timewise into training for the profession, that's a big, big loss. P5

As a recognized issue in the profession, the school began taking steps to build resilience in their students;

So we're starting to build in things around resilience and building up coping strategies to deal with failure. P2

#### Weight of responsibility for PhD students

It also became clear that these issues do not abate after undergraduate level. It was noted that it can be a very isolating experience, and a drastic contrast to the undergraduate experience. PhD students struggle with imposter syndrome, not having belief in their abilities and strengths to succeed;

…students often feel imposter syndrome … ‘you're not good enough, you shouldn't be here’. I think that's, thinking of mental health and wellbeing of students, I think that plays a big role as well. I think… it goes hand in hand with PhD students being, sort of, more on their own and isolated in terms of not being in a class and stuff and not being able to measure yourself against other people… I think they often feel like they're not doing a very good job when they are doing a very good job actually, and they're doing a very difficult one. And I think that plays into mental health. P6

PhD students feel pressure not only to live up to their own expectations but also the expectations of their supervisors and funders. This sense of responsibility can weigh on students;

PhD students are getting… messages that… research councils and funders will fund you as a researcher, your brain is what they're funding and so that's a huge responsibility. You're thinking ‘I'm bad’… it's kind of the force that people sort of self‐esteem and self‐worth. So you ran an experiment that was a cap, but you're not crap! That experiment is a pile of shite but you're fine', you know! P6

#### Educators taken for granted

I don't think staff are the priority for the university generally. I think they've made some efforts, priority, it depends what it's up against. P5

None of the academic staff interviewed felt academic staff's well‐being was a priority to the university. A common thread which appeared suggested that it is taken for granted that the staff will give as much as they can to their profession, which can lead to standards being set very high and well‐being suffering;

I think we expect our academics to do far too much to maintain a healthy lifestyle, healthy work‐life balance… that sort of issue which is much harder if you're not sort of, you know, white and male and don't have any kids and all of those sorts of things as well. So it has a knock on effect on people like diversity and stuff. P6

I want my lectures to be remembered and enjoyed and entertaining, and I want practicals… to run smoothly. If I'm designing a practical I want to deliver it in the best possible way…. I think generally… [I] put more pressure on myself than is put on me. P3

Additionally, there appeared to be a disconnect in what the university explicitly asked of the academic staff compared to what was actually achievable and that this had impacts on staff's well‐being;

The way the university could best look after staff's mental and psychological health… is to take a look at their workloads and make sure they're not asking them to fit square pegs into round holes. P5

#### New pressures for educators from COVID‐19

I think COVID has put a lot of strain on even the most resilient of people. P2

A number of participants expressed concern over their ability to connect with their students in the same way they had in the past, prior to video‐hybrid lectures. One participant expressed that they gauge how well students are grasping the content being taught. With the event of hybrid learning, this is not possible.

And that's the thing about COVID, the interactions that we used to have are gone, we kind of just jump in now. But I still do that with my students. We would have a chat and then get down to work. P1

Another participant highlighted that due to the hybrid model that was being suggested their workload had essentially doubled. Educators were being asked to prepare lessons for both e‐teaching and in‐person lectures;

…we're… being asked to… prepare classes for both online and face to face delivery, …that's effectively a double workload. If you have 3 lectures to give that's fine, but if you have 33 lectures to give and… as it's looking… I'd really like to take a couple of these weeks holidays that I have. So I think academia is one of those worlds in which it's understood that most of us, if we enjoy what we do we'll actually give an awful lot to that. Sometimes what that's lead to is the bar being set to what we should be giving which is maybe not quite realistic P5

The event of lockdown has caused schools and the university to pay closer attention to the well‐being of their staff. One school introduced a ‘no meetings on Friday’ policy. While other schools had good‐intentioned initiatives to offer as well, they seemed disconnected from what staff actually needed;

…we have a guy in our school who's called the student relationships manager… we've been getting a lot of inspirational messages recently, all these sorts of things. … we've got links to… resources that might help us meditate and, you know. And I think, while I'd love to meditate, it would be easier if I had… slightly more people to mark these assessments… P5

### Strengths‐based practices cultivating PsyCap in students

Because they're all individuals, and they've all got their unique strengths. P2

The data provided clear examples of strengths‐based approaches, which elicited psychological capital in some instances, in both students and educators (Table 2). The data also provided examples of these approaches mitigating some of the risk factors of students mentioned in ‘Pressures in Academia’. Evidence of the five principles of strengths‐based approaches could be seen in the following supraordinate themes; the educator as a guide and role model, cultivating excellence in students, the individualization of the learning experience, the mobility of learning and the value of resilience in light of COVID‐19. What is noteworthy is that no participant had prior knowledge of strengths‐based approaches, and each example provided for strength‐based approaches was unconsciously aligned with these approaches and, therefore, intrinsically motivated.

**TABLE 2:** Examples of codes for strengths‐based practices cultivating PsyCap in students

Role of reflection	Supportive environment
Creativity	Value in community
Relationship building	Strengths identification
Defining excellence	Value of novelty

#### The educator as role model and guide

And we are role models whether we like it or not, we are role models. P2

P1, P5 and P6 highlighted that students do not always know what they need to succeed and that the role of the educators is to guide them with information and exercises to facilitate the student's success.

The role of the educator is to act as a guide to students, implementing exercises and offering advice that the student may not fully understand or enjoy at the time, but knowing that it will serve them in their future;

…we have to… make sure they're not all driving themselves into the ground. And to make them realistic about what the requirements are; they have to pass, be safe and competent… and not to flay themselves too much. P5

A challenge can be offering advice, providing students with the insight to take necessary measures and precautions to aid success, only for the student to go another way or ignore it entirely;

Because you can see things coming… you can see that there will be an issue down the line…, but you can't do it for them. I think it's hard when you can see things not working out further down the line and you can see them coming but you can't stop them sometimes, um they just play out. Yeah, sometimes even with the best support that you can offer it still doesn't work. P6

This demonstrates that, as educators, they are not solely interested in the student's capacity to learn relevant course content, but that they are also invested in their students as individuals, that they truly care for their well‐being and success and want to take steps to aid their success and to help them thrive.

Every participant felt strongly that the student‐educator relationship can have a significant impact on a student, positive or negative. Two participants eluded to negative personal experiences from their days as a student where they were left feeling “stupid” and that they “…still have scars today”, demonstrating the extent of effect an educator can have on their students.

P6 recalled their own supervisor, from when they were completing their PhD, who demonstrated a healthy work–life balance and left a great positive impact on the participant. They instilled a positive approach to work, which is a value that this participant tries to imbue now in their PhD students;

If you have a supervisor saying ‘go home! What are you doing stuck here?’, who puts that value of work life balance and having a life outside of the lab… It's an excellent role model as they go through their career. P6

#### Cultivating excellence

… I think we can as an institution, we have control over what we choose to recognise as excellence… P6

A key takeaway was the power of choosing what excellence means and that the definition depends on the individual, not the institution. Participants acknowledged that there are specific standards that must be met, but that these standards are not so rigid that there is no space for the individual to thrive in their own definition of ‘excellence’.

Participants teaching styles reflected their hopes for their students to accomplish success, and being that person to provide support while pushing them to reach new heights;

… I'm very tough and demanding… in a supportive way… I'm happy with what they've done but I always think they can do better. P1

Participants also held optimism for the future. Understanding the accomplishments of their own generation, they wanted to lead the way for the next generation to do even better and push the limits of excellence even further;

I would like to think that the generations are coming after my own will do better than we've done. P1

#### The individualization of the learning experience

Just to see somebody sort of blossom… is just fantastic really. P3

It was very clear that the participants had a keen sense of their students as individuals and felt compelled to make them aware of that and to embrace it. In promoting their individuality, grounds for comparison become less effective and provide the student with the opportunity to develop themselves in their craft;

So it's not about trying to produce a particular type of writer, a particular style, it's about giving them confidence and skills to become the best writers that they can be as individuals. So I guess it's about working out where they're coming from, what their purposes are. So it's about building their confidence, and trying to give them the skills that they need, or refining the skills they need, to become the best writers they can be P4

By emphasizing individuality, it becomes harder to associate themselves as being an ‘outsider’ and different from peers, because everyone is seen as different. This approach helped in the abating that pressure high achievers felt when comparing themselves to their classmates;

…you will find there are different students who have different aptitude in different things to always remind them of that as well; that nobody is omnicompetent about everything. P5

While feeling isolated was a mentioned risk factor for students, individualizing the learning experience provides the opportunity for students to feel empowered in what they might view as the solo pursuit of their goals;

Through that project she kind of, she really found her strengths… P3

#### Mobility of learning

Participants reported that taking students out of the classroom to engage with new environments and professionals added great value to the learning experience. Some noted that it broadens their perspectives and holds the potential to engage in positive social interactions and build relationships, yet another means of abating feelings of isolation. One participant felt it was important that students be able to see the staff getting excited about topics outside the classroom and engaging with the physical field where they will likely one day have a career.

It… gives them an expanded sense of place and… the institution, that learning can take place out with the classroom… it broadens the notion of what a university can be… P4

Participant 4 found value in bringing students to the museums, relevant to the subject they teach. Engaging with custodians and experts brought an element of novelty and interdisciplinarity to the lesson. These interactions highlighted to students that learning does take place out of the university's walls;

…it can be a very… underrated activity to… get students outside out of the classroom and into the wild. P3

Taking students outside the classroom not only adds to the learning experience but also in relationship building with fellow students and with educators;

…it's very good for them to see the staff members getting excited about things. P3

…I think also it's a bonding thing. So there's a kind of social element, a… discovery… a sense of adventure to it, because you're venturing beyond the walls. P4

## DISCUSSION

**FIGURE 1:**
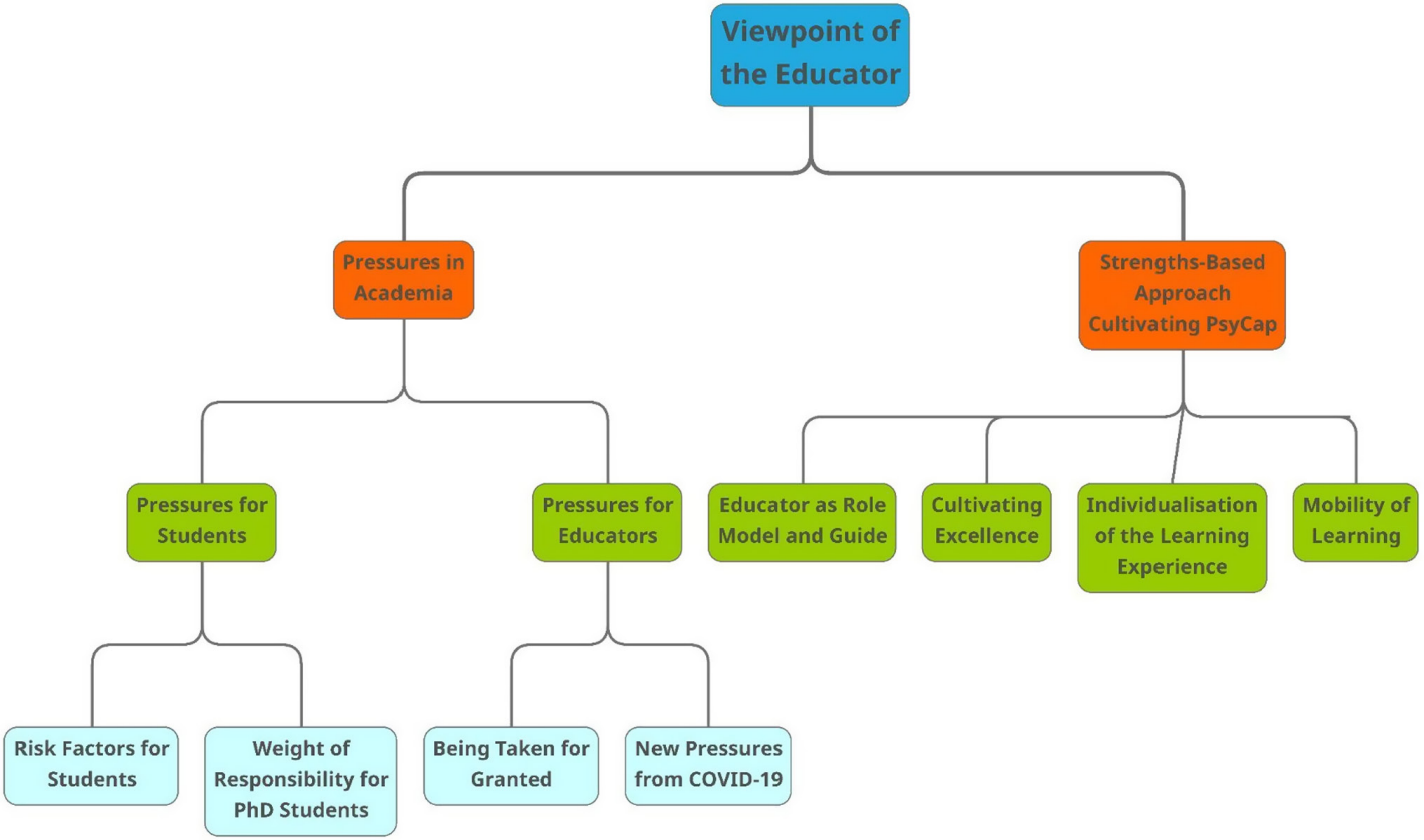
Theme map

This qualitative research aimed to identify strengths‐based practices used by award‐winning educators that cultivate psychological capital (PsyCap) in students and to explore pressures in academia (Figure 1). One of the main findings was that practices used by award‐winning educators can promote positive psychological capital in students and improve academic well‐being. Interviewing educators revealed that students' mental health is suffering in the current learning environment, which is in line with findings from Saleh et al. (2017). This study has shown that risk factors in students are born of lack of resilience and fear of failure. According to educators, students hold themselves to high standards and are hard on themselves when they do not meet these standards and often do not have the resilience necessary to ‘bounce back’ after experiencing failure. Moreover, comparing themselves to their peers can leave some feeling ‘stupid’, and in some instances even drop out of their course or career early. The subjectivity of failure means that the perception of failing is not necessarily tied to a grade, but tied to fulfilling the expectations a student sets for themselves.

However, risk factors in students identified by participants can be mitigated by the teaching methods and engagement styles used by educators, while fostering the four pillars of PsyCap. These teaching methods are in keeping with the five principles of strengths‐based approaches posited by Lopez and Louis (2009), even though participants had no previous experience with this approach. Participants emphasized the value of individualizing the learning experience for students, diminishing feelings of isolation and encouraging the development of individual student's strengths. These findings support the claims made by Carmona‐Halty et al. (2019), that positive relationships with educators cultivate positive well‐being in students. Additionally, exploring the mobility of learning contributes to the development of novel learning experiences and facilitates positive social interactions, increasing academic integration, retention rates and successful outcomes in academic performance (Sweet & Swayze, 2020).

Resilience is a key attribute to success in academia. The event of COVID‐19 and the first lockdown in which this study took place made this more apparent than ever. Not only were students facing exams and the typical stressors associated with university, but they were also contending with an unforeseen pandemic and overt life changes (Kim et al., 2021). The same can be said of the participants of this study, as they traversed new modalities of teaching while tending to their own well‐being. Participants highlighted the pressures they experience in their roles (e.g., demanding workloads, lack of training in handling sensitive information from personal tutees and maintaining high research outputs) and that resiliency is needed not only to thrive in academia but also to survive. As research suggests, resiliency and other PsyCap domains, such as hope and optimism, were signified as paramount in buffering symptoms of depression as well as in the subjective experience of well‐being (Prasath et al., 2021; Hagedorn et al., 2022). The findings of this study make plain the necessity of resilience within the academic sphere, not only for academic success but also for overall well‐being, hereto an under‐researched topic (Boullion et al., 2021; Martin, 2013).

Considering students' subjective conceptualizations of failure and excellence are key takeaways from this study to understand why students' mental health is suffering. While traditional standards of achieving high grades are perceived as excellence and poor grades as failure, everything in‐between and outside of course results are open to interpretation. The role of the educator in cultivating the individual‐based perception of excellence and the culture of the school can greatly influence these interpretations, which provide students with the skills to thrive in their university and professional career (Kahu & Nelson, 2018; Picton et al., 2018). Therein lies the value of the individualization of the learning experience, that students can separate themselves from their peers by recognizing their individual strengths and by seeing everyone as an individual without exclusion. Recognizing individual strengths is a powerful tool for students to carry with them through their careers and in their development of psychological capital. Therefore, it can be seen that PsyCap, which is cultivated through strengths‐based practices (i.e., educators acting as role models, offering guidance, and engaging in innovative and individualized‐approach to learning), plays a significant role in the mitigation of negative and risk factors in mental health and success in students.

Furthermore, the mobility of learning has the capacity to alleviate the imposter syndrome often felt by PhD students. By meeting experts in other fields of expertize and developing stronger relationships with peers and superiors, students can develop a sense of belonging in their subject area, and develop their psychological capital. These findings align with the suggestions put forward by Ramsey and Brown (2017), that seeking out mentors and other positive relationships has the potential to alleviate imposter syndrome. Moreover, the weight of responsibility felt by PhD students around funding and meeting the expectations of their supervisors can be lightened by developing increased levels of self‐efficacy. With supervisors incorporating strengths‐based advising into their supervision meetings, the student can gain confidence in their efficacy and take ownership of their research in an empowering way.

Lastly, this research project shows that academic staff are not receiving adequate support to fulfil their roles successfully without negatively affecting their own well‐being. Educators feel unappreciated for the work they do and are overworked, with some even requiring leave for health reasons. There is extensive literature indicating the high rates of burnout among faculty members in academia, which were heightened during the COVID‐19 pandemic (Fowler, 2015; Leal Filho et al., 2021; Parte & Herrador‐Alcaide, 2021; Taylor & Frechette, 2022; van der Ross et al., 2022; Watts & Robertson, 2011). As such, these results demand that emphasis be placed on the development of psychological capital in educators by universities. Currently, educators are making sure they meet the standards set by the university and by themselves to ensure excellence and a supportive environment for students, while their own well‐being pays the cost. This must be addressed, or the standards that enable students to thrive will not be achievable because their educators are suffering.

The number of participants was sufficient that variety was available in the data without crowding the results, and allowed for a rich, in‐depth analysis of the data. Additionally, using the six‐step approach to thematic analysis by Braun and Clark (2006) ensured rigour in the analysis and enabled the exploration of subtleties and complexity that is challenging to achieve in quantitative research. As transcription software was not used, the researchers maintained a close connection with the data set, allowing for depth of insight and analysis. Maintaining a record of the research team's personal reactions and reflections aided the validity of the research.

### Strengths & limitations

As this study took place during the COVID‐19 lockdown, it was evident that participants' opinions were influenced by their current circumstances. Nevertheless, the research design proved to be durable and flexible in light of the lockdown measures for COVID‐19. The in‐depth interviews were an effective choice of data collection as they allowed for flexibility in questioning and gleaned rich and insightful qualitative data. Video calling made it possible to read social cues, such as facial expressions and body language, aiding in the flow of the conversation. Opting to interview award‐winning educators from the University of Edinburgh further allowed for prompt recruitment and homogeneity of the sample.

However, the addition of a student population would have provided a more complete picture of the research aims. Moreover, it would have provided the opportunity for students to provide insight into their personal experiences with educators and the aspects of teaching practices that they enjoy. However, given the constraints of the study and taking into account previous research in this area (Carmona‐Halty et al., 2019; Datu & Valdez, 2015; Siu et al., 2013) the understanding of the educators perspective added more to the current knowledge base.

### Future implications

Future research should explore the efficiency of strengths‐based approaches through an intervention study in comparison to teaching as usual to explore the potential of developing psychological capital in students in a UK university setting. Building on research by Soria and Stubblefield (2015), the StrengthsFinder 2.0® assessment could be completed by incoming first‐year students to discover their top five talents. Students could also be encouraged to take the Psychological Capital Questionnaire (Avey et al., 2011). Both assessments should be administered prior to matriculation and again at the end of their first year to allow for comparison.

## CONCLUSION

This study has highlighted the potential merits of using strengths‐based principles to mitigate the negative factors experienced by students, and the capacity to aid the cultivation of. The gap in research focusing on well‐being among educators was highlighted, and pressures that educators feel in their roles were explored; this included feeling taken for granted and held to excessive measures of excellence set by themselves and the university. While more research is needed to establish the benefits of strengths‐based education, its merits can be seen in the cultivation of PsyCap in students. The benefits of these positive psychological concepts can have a worthwhile effect on the well‐being of students *and* educators.

## AUTHOR CONTRIBUTIONS

**Roseanne Morris:** Conceptualization; data curation; formal analysis; investigation; methodology; project administration; validation; writing – original draft; writing – review and editing. **Mark Hoelterhoff:** Conceptualization; formal analysis; supervision; writing – review and editing. **Georgios Argyros:** Validation; writing – review and editing.

## CONFLICT OF INTEREST

The authors declare that there is no conflict of interest.

## Data Availability

The data that support the findings of this study are available on request from the corresponding author. The data are not publicly available due to privacy or ethical restrictions.
